# What is an emergency? Neonicotinoids and emergency situations in plant protection in the EU

**DOI:** 10.1007/s13280-022-01703-5

**Published:** 2022-01-29

**Authors:** Yaffa Epstein, Guillaume Chapron, François Verheggen

**Affiliations:** 1 Swedish Collegium for Advanced Study and Department of Law, Uppsala University, Östra Ågatan 19, 753 22 Uppsala, Sweden; 2 Department of Ecology, Swedish University of Agricultural Sciences, Uppsala, Sweden; 3 Gembloux Agro-Bio Tech, University of Liège, Agro-Bio Tech Passage des Déportés 2, 5030 Gembloux, Belgium; 4 Inst. För Ekologi, Grimsö forskningsstation, 739 93 Riddarhyttan, Sweden

**Keywords:** Bees, Biodiversity, Environmental law, EU law, Pesticides, Pollinators

## Abstract

Actions potentially harmful to the environment that are otherwise illegal are sometimes permitted in cases of emergency. How to define an emergency can therefore be both controversial and highly consequential. In this article, we explore one such contemporary controversy: when the use of neonicotinoid pesticides, banned in the EU, can nevertheless be granted an emergency authorization. We analyse several questions, currently before the EU Court of Justice in the ongoing *Pesticide Action Network Europe and Others* case, that will determine the scope of an “emergency” in the context of derogating from the Pesticide Regulation, and that may impact how “emergencies” are defined in other legal contexts. We argue that the circumstances do not support a legal finding that emergency authorization is justified in this case, and that, in general, “emergencies” must be narrowly defined when justifying measures that involve risks to human health and the environment.

## Introduction

What legally constitutes an emergency is a question that arises in a variety of contexts. Many laws include provisions that are meant to allow for flexibility to deal with emergencies. Some legal emergency measures seek to protect human health or the environment by placing limitations on other rights or interests, for example, restricting freedom of movement to address the COVID-19 pandemic (Canestrini 2020). Other types of emergency measures allow for risks to human health and the environment in order to protect other interests, such as economic interests.

The European Union’s Plant Protection Product Regulation, Regulation 1107/2009 (European Parliament & Council of the European Union 2009a) (“Pesticide Regulation”) is “underpinned by the precautionary principle” (Pesticide Regulation, article 1) and seeks to protect human and animal health and the environment by disallowing the use of pesticides unless it has been demonstrated that the pesticide is not harmful. It allows for exceptions to be made in certain circumstances; it has both emergency provisions that respond to threats to health and the environment, and emergency provisions that respond to threats to other interests. In one set of measures for responding to emergencies, the Pesticide Regulation allows action to be taken to suspend the use of an approved pesticide if it becomes clear that the pesticide “is likely to constitute a serious risk to human or animal health or the environment” (Pesticide Regulation, articles 69–71). That is, these provisions apply when the Regulation’s precautionary requirements have been insufficient to ensure safety. The other set of emergency measures allows exceptions to be made to the Regulation’s precautionary requirements—emergency authorizations to allow the use of unapproved pesticides despite potential risks to health and the environment—to be made in “emergency situations in plant protection” (Pesticide Regulation, article 53).

Emergency authorizations have been widely used by Member States, particularly for neonicotinoids, a controversial class of pesticides. This practice has been challenged in *Pesticide Action Network Europe and Others,* Case C-162/21 (*Pesticide Action Network*), a case that is likely to have far-reaching repercussions for both EU agriculture and pollinator conservation (Epstein et al. 2021). In this article, we examine the delineation of emergency in this legal context, that is, the possibility for emergency pesticide authorizations, and draw conclusions relevant for the analysis of emergencies in broader legal contexts, most directly EU environmental and health law.

## Neonicotinoids are banned in the EU, yet widely used

Neonicotinoid pesticides are controversial because of their potential to harm non-target species, particularly pollinating insects. Several studies have also suggested that neonicotinoids can negatively impact insect-eating birds (Hallmann et al. 2014), as well as mammals, including humans, that might be directly exposed to neonicotinoids through, for example, consumption of improperly buried treated seeds or pesticide dusts released by the seeds (Thompson et al. 2020). Following the demonstration of the adverse effects of neonicotinoids on bees (Whitehorn et al. 2012), the European Commission limited the use of the neonicotinoid pesticides clothianidin, thiamethoxam and imidacloprid outside of permanent greenhouses in 2013 (European Commission 2013).

The use of neonicotinoid treatments on beet seeds continued to be allowed because it was considered lower risk. Beets are harvested before flowering, and bees are therefore less likely to be exposed to their pollen. However, residues left in the soil after harvest may be transmitted in succeeding flowering crops (Viric Gasparic et al. 2020). After an evaluation, the bans were strengthened in 2018 to include a prohibition of all use outside of permanent greenhouses (European Food Safety Authority 2020; e.g., European Commission 2018). The pesticide manufacturer Bayer CropScience challenged the bans in 2013, but both the challenge and an appeal were rejected by the EU courts, with a final decision upholding their legality handed down in 2021 (Bayer CropScience AG and Bayer AG v European Commission, Case C-499/18 P).

However, despite the ban, and despite additional EU legislation calling for a reduction in pesticide use (European Parliament & Council of the European Union 2009b), the use of neonicotinoids remains widespread in the EU through the use of the aforementioned emergency derogation provision. Several Member States, including Austria, Belgium, Croatia, Denmark, Finland, France, Lithuania, Poland, Romania, Slovakia and Spain, have continued to allow the “emergency use” of banned neonicotinoids for major crops, especially seed treatments for sugar beets (European Food Safety Authority 2020).

The reason that neonicotinoids continue to be used is that they are a relatively cheap and effective means of protecting crops from insects, particularly the aphids that are a vector of viral infections—virus yellows—that can infect sugar beet crops. Even small numbers of aphids can harm sugar beet crops because they spread plant viruses. Plant diseases like virus yellows reduce the yield and sugar content of beets by damaging leaves and thus reducing photosynthesis. These viruses are widespread in Europe, and without prevention measures, non-negligible levels of crop loss are very likely. According to Hauer et al. (2017), the use of neonicotinoid-treated seeds increased sugar yields in areas with pests by an average of 7% compared with no insecticide use. However, other studies suggest that crop loss could be significantly higher (Hossain et al. 2021). Because of the widespread use of neonicotinoids in Europe prior to 2019, there have thus far been few studies that can show the likely crop loss due to natural infection in the field. Losses are, however, likely to vary greatly by region and by year.^1^

Seeds are coated in the product, which then circulates through the plants’ sap, protecting young plants for about 12 weeks. Other, non-banned, insecticides exist, but have drawbacks compared with neonicotinoid seed treatments (Elbert et al. 2008). Multiple foliar applications are required to have the same effect, which is more expensive, more time consuming, and requires the use of more pesticide. Because foliar pesticides act by contact, moreover, they often fail to kill aphids hiding on the interior face of leaves (ANSES 2021). Whether these advantages are sufficient to justify emergency authorization of a pesticide that has been determined to cause an unacceptable risk to the environment is now being questioned.

In the request for a preliminary ruling *Pesticide Action Network*, the Belgian Council of State has asked the Court of Justice of the European Union (Court of Justice) to interpret what constitutes an emergency situation in plant protection. After the Commission issued its 2018 regulations strengthening the ban on neonicotinoids, Belgium granted six emergency authorizations for the spring of 2019 allowing for the marketing and treatment of seeds with thiamethoxam and clothianidin. These decisions were appealed by two anti-pesticide non-governmental organizations and a bee keeper to the Belgian Council of State, which has in turn asked the Court of Justice to help it determine whether the emergency authorizations were permissible under the Pesticide Regulation. In the next sections, we address aspects of the questions in this case that help interpret what constitutes an emergency situation in plant protection, and more broadly, what defines an emergency that can justify derogating from a law that protects health or the environment.

## Can preventive measures be emergency measures? Foreseeability, uncertainty and emergency

In dispute in *Pesticide Action Network* is whether measures enacted long before the presence of a danger, here coating seeds in pesticides, and sowing such seeds, can be considered emergency measures. The answer depends on how one defines emergency. In this section, we consider whether emergency situations in plant protection can be foreseeable, common or predictable, and to what extent they must be. We then discuss whether the fact that seed treatments are used prior to an existing threat makes them less acceptable as emergency measures than other pest prevention methods.

The ordinary meaning of “emergency” generally requires some degree of surprise and urgency. The Oxford Dictionary of English, for instance, defines it as “a serious, unexpected, and often dangerous situation requiring immediate action”. What a word means in a law can of course be different from what it means in the dictionary. The Pesticide Regulation does not, however, explicitly define emergency in the context of emergency authorizations. The relevant article, Article 53, is titled “Emergency situations in plant protection”. The first subsection under that title reads: “By way of derogation..., in special circumstances a Member State may authorise, for a period not exceeding 120 days, the placing on the market of plant protection products, for limited and controlled use, where such a measure appears necessary because of a danger which cannot be contained by any other reasonable means”. The relationship between the title and the operational part of the article is somewhat unclear. It might be argued that the emergency situation referenced in the title is meant to be defined as a circumstance in which a danger exists which cannot be contained by other means.^2^ This definition of emergency would leave open the possibility that Europe could be in a continual and predictable state of aphid emergency, if aphids were considered to constitute such a danger.

However, the argument that the use of the word “emergency” in the article title is intended to indicate that the "special circumstances" referred to in the operational part should be unforeseen is supported by an examination of the Pesticide Regulation’s 1991 predecessor regulation. In that earlier law, the corresponding provision was included in a longer article on transitional measures and derogations and did not have its own title. The phrase “emergency situations” was thus not used, but the operational part allowed derogation when “necessary because of an *unforeseeable* danger which cannot be contained by other means” (European Parliament & Council of the European Union 1991) (emphasis added). The word “unforeseeable” seems to be replaced by the term “emergency situation” in the current article title. While there are arguments to be made in both directions, this earlier phrasing, combined with the common meaning of “emergency”, suggests that emergency situations envisioned by the current Pesticide Regulation are not only unavoidably risky to plants, but also to some degree unforeseeable, or at least unexpected.

How a word is used in one context in a law can indicate how it meant to be understood in another context in the same law. Additional but limited support for the argument that emergency authorizations are meant to be restricted to unexpected situations can be found in the Pesticide Directive’s other emergency provisions. In its Chapter IX, “Emergencies”, the Pesticide Regulation outlines the “emergency measures” to be taken when it is clear that a previously approved pesticide is “likely to constitute a serious risk to human or animal health or the environment…”. Here, that an emergency requires a risk of harm is explicit, and the unforeseeability of the risk is again implicit: had the risks to health or the environment been foreseen, the pesticide would not have met the criteria for approval in the first place. In both the Regulation’s uses of the term “emergency”, then, a requirement for unforeseeability does seem implied.

More attenuated but still informative is how a word is used in other laws. Other areas of EU law dealing with different types of emergency situation often specify that some element of the unforeseen is required in order to derogate. For example, the Directive on Returning Illegally Staying Third-Country Nationals also titles a derogation provision “Emergency situations”. This provision, Article 18, says “In situations where an exceptionally large number of third-country nationals to be returned places an unforeseen heavy burden on the capacity of the detention facilities of a Member State or on its administrative or judicial staff, such a Member State may, as long as the exceptional situation persists, decide to allow for periods for judicial review longer than those provided for…”. (European Parliament and Council 2008). Here it is made explicit that the circumstances leading to the situation must be “unforeseen”.

But even if it is determined that foreseeable threats can constitute emergencies, there must be a limit on how common or predictable the threat can be. As argued by Belgium in its emergency authorizations, “severe outbreaks of virus yellows are a near certainty”.^3^ This certainty seems to negate the idea that the threat of aphid infestation and virus yellows is a “special circumstance” that can allow for emergency derogation. The point of the Pesticide Regulation and the ban on neonicotinoids is that states must use pest control solutions that have been determined not to pose a threat to the health of humans, animals or the environment. If common situations could be construed as special circumstances justifying exception from the regulation, the regulation would have little impact.

A similar line of reasoning can be found in the Passenger Compensation Regulation, which exempts carriers from the requirement to compensate for delayed or cancelled flights in “extraordinary circumstances which could not have been avoided even if all reasonable measures had been taken” (European Parliament and Council 2004). The phrase “extraordinary circumstances” was interpreted, in the case *Transport Aéreos Portugueses* (Case C-74/19), to include the need to divert a plane to offboard an unruly passenger, if and only if there had not been warning signs of the unruly behaviour before or during boarding. Therefore, the foreseeability of the negative outcome negated the existence of “extraordinary circumstances”, perhaps as the foreseeability of crop loss in the absence of alternate means of pest control may be found to negate the existence of special circumstances. The difference between the “extraordinary circumstances” of the flight compensation regulation and the “special circumstances” of the Pesticide Regulation are unclear, but some parallels are warranted. In any case, when emergency circumstances are used to justify derogating from a protective law, they must be narrowly construed.

Clearly, however, derogation must also require a reasonable degree of likelihood that damage will occur absent the derogation—derogations must be “necessary”. In this case, several things are not in dispute. First, the emergency situation has not yet occurred at the moment of pesticide application: there are not aphids present when the coated-seeds are planted. It is, as Belgium states in the disputed applications, not “known in advance where severe pest outbreaks might occur”.^4^ Further, as 99% of the Belgian beet production currently uses neonicotinoids, there is limited data on the impact of not using neonicotinoids on Belgian beet production. It is also undisputed that, absent the use of neonicotinoids, some degree of crop loss is likely to occur somewhere, though the precise location and extent cannot be known in advance. In order to justify using a pesticide that has been determined to pose an environmental risk, the likelihood that other dangers will be averted must be more than speculative.

Requirements for emergency plans in various laws also imply that accidents and emergencies are to some degree expected to occur and that it is possible to act to mitigate their impact. The Directive on the Control of Major-Accident Hazards Involving Dangerous Substances (Dangerous Substance Accident Directive), for example, requires that establishments where dangerous substances are present in significant quantities have an emergency plan for limiting the effects of “incidents” and implementing measures to protect human health and the environment after major accidents (European Parliament & Council 2012). So, according to this Directive, emergency measures are those taken after an emergency has occurred, but they nevertheless can and must be planned for. Emergency measures are also contrasted with preventive measures in some situations. According to the Regulation Concerning Measures to Safeguard the Security of Gas Supply (Gas Supply Security Regulation), Member States are required to establish both preventive actions plans and emergency plans to deal potential disruptions of gas supply (European Parliament and Council 2017). As in the Dangerous Substance Accident Directive, emergency measures are reactive to a disruption that is already ongoing, while preventive measure are obviously taken prior to an emergency with the goal of preventing that emergency from happening. A common theme in these laws is that emergencies are unpredictable. Still, where possible, preventive measures that comply with applicable laws should be taken to avert their impact.

Seed treatments are preventive rather than responsive; they anticipate the danger before it occurs. The same might also be said for foliar treatments and other means of pest control. As a very small number of aphids can be responsible for a significant virus spread, applying pesticides after their presence is detected is too late to prevent crop damage. Insect forecasts, for instance by agricultural associations, allow agricultural producers to determine when to apply foliar pesticides before damage occurs (Olatinwo and Hoogenboom 2014).

There is, however, a difference in the urgency and certainty of the adverse event. When seeds are planted, it is not known whether or the extent to which that particular area will be impacted by aphids, though it is very likely that some instance of pest will occur at some time. Foliar treatments are applied when experts advise that an outbreak of aphids or viruses is immanent based on monitoring and forecasting—the extent of harm caused by doing nothing is yet unclear, but significantly more certain than at the planting stage. These differences in degrees of uncertainty lead to a conclusion that seed treatments should be considered differently than other applications, and weighs against their being considered appropriate for use in emergency derogations.

## Should banned pesticides be treated differently than non-approved pesticides in the derogation context?

The Pesticide Directive's provision on emergency situations explicitly allows for derogation in certain circumstances to permit the use of non-approved pesticides. It is silent on whether derogations can be granted for substances that have previously been approved, but later were explicitly banned after an evaluation of their potential harm. The neonicotinoids in this case had been banned outside of permanent greenhouses after an evaluation of their risks to bees, in separate implementing regulations that do not specify a possibility for derogation (European Commission 2018).

The fact that these substances have been determined to pose an unacceptable risk to bees should factor into the evaluation of whether the emergency authorization is justified. In some ways, it is easier to weigh the risk of granting the derogation against the risk of not granting it when the danger is already known. However, emergency derogation is most justifiable when it appears likely a substance or use will be approved if there was time for the process to be completed. This seems in line with the European Commission’s view expressed in its guidance document on emergency authorizations, which states “…emergencies demand quick and effective responses that often cannot await the outcome of the regular authorization process…” (European Commission 2021). When it comes to banned substances, it is already known that the substance is not likely to be approved in the future. Approval of emergency authorizations for a substance that has already been judged by the European Commission to pose an unacceptable risk to bees should therefore be very limited.

## What is the role of cost in evaluating if an alternate means of plant protection is reasonable?

The preamble to the Pesticide Regulation includes the clause “The purpose of this Regulation is to ensure a high level of protection of both human and animal health and the environment and at the same time to safeguard the competitiveness of Community agriculture”. A question put to the Court of Justice by the Belgian Council of State is whether the states should consider “safeguarding the competitiveness of Community agriculture” to be of equal importance to the high level of protection for health and the environment in deciding whether other alternatives for protecting crops other than derogation are “reasonable”. That is, whether the fact that using available approved methods of controlling pests could make the sugar more expensive than sugar produced elsewhere and therefore potentially non-competitive in the absence of other support, justified allowing the use of neonicotinoids or other non-approved pesticides.

This question recalls the decision by the Court of Justice in its decision in the 2000 case *First Corporate Shipping* (C-371/98). In that case, the Court held that although the Habitats Directive (Council of the European Communities 1992), which aims to protect biodiversity in the EU, states that measures taken “shall take account of economic, social and cultural requirements and regional and local characteristics”, these requirements and characteristics did not constitute additional reasons to make exceptions to that Directive’s protective requirements. Similarly in this case, the competitiveness of the EU’s agriculture, which was considered in formulating the requirements of the Pesticide Regulation, is not likely to constitute a basis for derogation.

The Pesticide Regulation presumably does intend to allow the cost of not derogating to be a factor, however, in the determination that an emergency exists. It is hard to imagine an emergency situation in plant protection that does not involve potential crop loss. Given the common market and possibility to purchase food from external sources, the risk of crop loss is largely financial, rather than a threat to human food security or the like. However, the Pesticide Regulation is explicitly a precautionary law. As argued by (Leonelli 2020), the “precautionary approach to risk management can hardly be cost–benefit effective”. These environmental laws exist to ensure that health and the environment are prioritized over profits. While economic impact is likely intended to be a factor in weighing whether derogation is justified, the precautionary intent of the Pesticide Regulation must guide the evaluation.

## Discussion

The emergency use of neonicotinoid-treated seeds is not legally justified in the *Pesticide Action Network* case, even though absent their use, pest control in beet fields will likely be more difficult. First, the use of treated seeds is a prophylactic measure taken before aphids are present and before it is known whether aphids will be present. At that point, it is possible and necessary to plan for alternate, permissible, means of pest control. Second, “special circumstances” do not seem to exist at the time that the measures are taken, because the threat of aphids is both constant and unpredictable. Aphids are always present in Europe and measures to control them must be taken annually, and therefore there are no special circumstances justifying emergency measures. At the time of planting, it is unknown whether aphids will be present in any particular field, also leading to the conclusion that there is no “special circumstance” at the time the pesticide is used. Further weighing against the appropriateness of these emergency authorizations is the fact that neonicotinoids are not merely unapproved, they have been banned due to known risks to bees. And while the threat of aphids to crops is a danger that factors into the permissibility of an emergency authorization, it does not negate the prerequisite that there must exist special, emergency, circumstances. In sum, Member States must use the alternate methods of pest control that are currently available, and should work to develop improved alternate methods (Jactel et al. 2019).

Examining the *Pesticide Action Network* case illustrates one situation where several factors combine to negate the existence of an emergency. It can also help illustrate the difficulties in delimiting emergency situations in plant protection and other administrative situations where emergency derogation might be allowed despite risks to human health, animals or the environment. The definition that we have argued for sounds somewhat paradoxical on its surface: an emergency situation can be neither certain nor uncertain, and must be foreseeable and unforeseeable. By analogy to other types of emergencies, however, it becomes apparent that this must be true.

For example, there is a high probability that a hurricane will occur somewhere in Florida in any given year. For this reason, Florida has adopted a building code requiring particular building standards in coastal areas, to reduce damage from high winds (Simmons et al. 2019). These measures, taken with the knowledge that an emergency is likely to occur at some time in the future, are preventive and not emergency measures. They do not excuse the need to comply with other building standards. When expert forecasting indicates a likelihood that a danger exists in a particular location in the immediate future, emergency measures, such as requiring evacuations, can be taken. After the damage has occurred, a state of emergency may be declared to direct funds to recovery efforts. These measures taken after the fact to facilitate a return to normalcy are also emergency measures.

On the other hand, a hazard with a high probability of recurrence might be argued to be permanent state of emergency, that is, because aphids are continually present in Europe, it might be argued that Europe is in a constant state of aphid emergency. This is essentially the position taken by some governments in the wake of terrorist attacks (Neocleous 2006). Terrorist attacks share some similarities with aphid infestations and hurricanes in that we do not know exactly when or where they will occur, but know that there is a high likelihood they will occur sometime and somewhere. By framing this as a long-term state of emergency rather than the status quo, governments justify the long-term or recurring use of measures that infringe on human rights, such as detaining suspects without trial (Neocleous 2006). While this use of emergency measures is a sort of precedent for a broad definition of emergency, it is one that is widely criticized (Neocleous 2006). Further, while it is sometimes asserted that these extended limitations on individual rights are necessary to protect the collective safety, in the case of neonicotinoids, the risk is to the collective safety, and the interests to be protected are the economic interests of the beet industry.

The strongest argument that there is an emergency is the claimed financial emergency to the beet industry. Beet industry representatives argue that the potential increased costs and yield losses would make their industry unable to compete with cheaper sources of sugar from outside the EU, potentially leading to lost income and jobs. On the other hand, the cumulative risks to bees could also lead to financial losses many times greater than the cost of financially compensating the beet industry for yield losses (Porto et al. 2020).

As argued by legal scholar Cass Sunstein, no measure is truly precautionary because every action and non-action carry some type of risk (Sunstein 2005). Banning neonicotinoids decreases risks to pollinators but increases risks to beet production. However, in EU administrative law, the precautionary principle has a specific, albeit contested, meaning. When laws, like the Pesticide Regulation, have precautionary aims of protecting health or the environment, action may be taken to prevent harm even when the potential harm is uncertain, and even when other interests are harmed. This was essentially the result in the aforementioned 2021 *Bayer CropScience* case, in which the Court of Justice upheld the neonicotinoid ban despite a lack of scientific certainty and despite the harm to the financial interests of the appellants. However, when derogating from such precautionary regulations, the same is not true. Possibilities for derogations must be narrowly construed so as not to undermine the intent of the law. There must be a higher level of certainty about the harm to be prevented to justify taking risks to human health and the environment. When weighing harm to these non-financially compensable interests against harm to financially compensable interests, there is a strong presumption that alternative, financial remedies should be considered another reasonable means for responding to the potential danger, in place of emergency authorizations.

## Footnotes

1 The French industry association L'Institut Technique de la Betterave (Technical Institute for the Beetroot) claims losses vary with time an place, for example reporting that different regions of France faced losses between 19 and 36% in 2017. See https://www.itbfr.org/tous-les-articles/article/news/f-a-q-tout-savoir-sur-la-jaunisse/ (accessed 17 September 2021).

2 This is essentially the argument made by a Belgian beet growers association that filed interventions in support of Belgium in *Pesticide Action Network*, see Summary of the request for a preliminary ruling at paragraph 57.

3 E.g. Notification of an Emergency Authorization Issued by Belgium, BE-Be-2018-30 at Box 15, available at https://ec.europa.eu/food/plant/pesticides/eu-pesticides-database/ppp/rest/pppapi/emergAuthAPI/authorisationsPDF/1259.

4 Ibid.
